# Antiangiogenic therapy for advanced renal cell carcinoma: Management of treatment-related toxicities

**DOI:** 10.1007/s10637-012-9796-8

**Published:** 2012-02-12

**Authors:** Roger B. Cohen, Stéphane Oudard

**Affiliations:** 1 Division of Hematology/Oncology, University of Pennsylvania, 16 Penn Tower, 3400 Spruce Street, Philadelphia, PA 19104 USA; 2 Medical Oncology Department, Georges Pompidou European Hospital, 20 Rue Leblanc, Paris, France

**Keywords:** Axitinib, Renal cell carcinoma, Tyrosine kinase inhibitor, Toxicity, Adverse events

## Abstract

Treatment of metastatic renal cell carcinoma (mRCC) has evolved rapidly over the last two decades as major pathways involved in pathogenesis have been elucidated. These include the vascular endothelial growth factor (VEGF) axis and mammalian target of rapamycin (mTOR). Therapies targeting the VEGF pathway include bevacizumab, sorafenib, sunitinib, pazopanib, and axitinib, whereas temsirolimus and everolimus inhibit the mTOR pathway. All of these novel therapies—VEGF and mTOR inhibitors—are associated with a variety of unique toxicities, some of which may necessitate expert medical management, treatment interruption, or dose reduction. Common adverse events with newer drugs include hypertension, skin reactions, gastrointestinal disturbances, thyroid dysfunction, and fatigue. Skilled management of these toxicities is vital to ensure optimal therapeutic dosing and maximize patient outcomes, including improved survival and quality of life. This review describes and compares the toxicity profiles of novel molecularly targeted agents used in the treatment of mRCC and presents guidance on how best to prevent and manage treatment-related toxicities. Particular attention is given to axitinib, the newest agent to enter the armamentarium. Axitinib is a second-generation receptor tyrosine kinase inhibitor with potent VEGF receptor inhibition that provides durable responses and superior progression-free survival in advanced RCC compared with sorafenib.

## Introduction

The incidence of kidney cancer has been increasing worldwide, accounting for approximately 2% of all cancers (excluding non-melanoma skin cancer) [1]. In 2010, 287,421 new cases and 122,302 deaths were estimated and, by 2015, 325,433 new cases and 138,629 deaths are expected to occur [2]. Incidence and mortality rates were highest for men in more developed areas, where kidney cancer comprised 4% of all cancers [3]. Although 5-year survival rates approximate 85% for patients with localized renal cell carcinoma (RCC) (the most common type of kidney cancer), patients with advanced disease have a 5-year survival rate of only 10% [4]. Nearly half of patients with RCC eventually develop advanced disease including 30% of patients initially presenting with advanced disease and another 20–30% with early-stage disease who relapse after nephrectomy [4]. Kidney cancer subtypes include clear cell RCC (85%) and the less common non-clear cell cancers, including papillary, collecting duct, and chromophobe RCC [5].

Chemotherapy and hormonal therapy are generally ineffective in treating kidney cancer; immunotherapy with high-dose interleukin-2 or interferon-alfa (INF-α) is effective in some patients, particularly those with good performance status [6]. These agents are associated with low response rates (<15%) and significant toxicities, which often limit their use and affect patient quality of life (QoL) [7].

## Targeted pathways in advanced RCC

Research on the molecular pathobiology of advanced RCC has identified the vascular endothelial growth factor (VEGF)/VEGF receptor (VEGFR) axis and the phosphatidylinositol-3-kinase–protein kinase B/mammalian target of rapamycin (mTOR) pathway lying downstream (the “angiogenesis axis”) as clinically relevant targets [8–10]. Transcription of vasculogenic mediators including VEGF and platelet-derived growth factor (PDGF) is promoted by stressors such as hypoxia, which is a strong signal for cancer angiogenesis. Angiogenesis in RCC is believed to be highly dependent on VEGF, due mainly to the high frequency of germline mutations in the von Hippel-Lindau (*VHL*) tumor suppressor gene. *VHL* mutations result in constitutive stabilization of the transcription factors HIF-1α and HIF-2α, which activate VEGF genes, thereby promoting angiogenesis [11]. Approximately 40% to 60% of patients with VHL disease, an autosomal dominant familial cancer disorder, develop clear cell RCC [11–13]. *VHL* mutation is also associated with approximately 50% of nonhereditary (sporadic) clear cell RCC.

The VEGF/VEGFR axis plays a critical role in tumor growth and survival [9]. Inhibitors of this pathway are thought to exert their effects by inducing apoptosis, cytostasis, and restrictive effects on tumor vasculature [10]. VEGF-targeted agents include the monoclonal antibody bevacizumab which neutralizes VEGF itself, and receptor tyrosine kinase inhibitors (TKIs) such as sorafenib, sunitinib, pazopanib, and axitinib. These agents target the VEGFRs, as do additional TKIs in ongoing clinical development, with effects that extend beyond the VEGFRs [14, 15].

The new wave of US Food and Drug Administration–approved molecularly targeted antiangiogenic agents has largely supplanted cytokines as first- and second-line therapy for metastatic RCC (mRCC). Second-generation molecularly targeted therapies in development include axitinib (a selective and highly potent VEGFR inhibitor); tivozanib and cediranib (also VEGFR inhibitors); brivanib (inhibitor of VEGFR and fibroblast growth factor receptor); motesanib (inhibitor of VEGFR, PDGF receptor, and c-Kit); XL184 (inhibitor of VEGFR-2, MET, and RET); and VEGF TRAP (novel inhibitor of VEGF-A).

Timely and appropriate management of treatment-related toxicities is vital in order to deliver therapy safely and optimally. This review describes and compares the toxicity profiles of antiangiogenic agents used in mRCC. Particular attention is devoted to axitinib, an antiangiogenic multi-targeted TKI in active clinical development for mRCC. Guidelines for preventing and managing treatment-related toxicities of axitinib are presented, which also have general relevance to all of the small-molecule angiogenesis inhibitors.

## Efficacy of new antiangiogenic agents in pivotal clinical trials

Findings from key clinical trials of approved antiangiogenic agents (sorafenib, sunitinib, bevacizumab, and pazopanib) in advanced RCC have reported consistent prolongation of progression-free survival (PFS) and, in some cases, overall survival (OS) in both treatment-naïve and previously treated patients (Table 1).

**Table 1:** Overview of efficacy of targeted therapies for mRCC

	Bevacizumab + IFN-α^a^	Sorafenib^b^	Sunitinib^a^	Pazopanib^b^	Axitinib^a^
Treatment-naïve					
Study 1	vs IFN	vs IFN	vs IFN	vs PBO	
	*N*=649 [23]	*N*=189 [79]	*N*=750 [43]	*N*=435 [55]	
PFS (mo)	11 vs 5	5.7 vs 5.6	11 vs 5	11.1 vs 2.8	
	HR = 0.63; CI, 0.52–0.75; *P*=0.0001		HR = 0.42; CI, 0.32–0.54; *P*<0.001	HR = 0.46; CI, 0.34–0.62; *P*<0.0001	
ORR (CR + PR)	31 vs 6%^c^	5.2 vs 8.7%^d^	37%*		
OS (mo)	23.3 vs 21.3 [78]	NR	26.4 vs 21.8 [28]	32%^d^ NR	
	HR = 0.86; CI, 0.72–1.04; *P*<0.1291		HR = 0.821; CI, 0.673–1.001; *P*=0.051		
Study 2	vs IFN			OL	
	*N*=732 [80]			*N*=155 [81]	
PFS (mo)	8.5 vs 5.2			NR	
ORR (CR + PR)	25.5 vs 13.1			34%	
OS (mo)	18.3 vs 17.4 [25]			NR	
Cytokine failure					
Study 1	vs PBO	vs PBO	OL	vs PBO	OL
	*N*=116 [82]	*N*=903 [30]	*N*=106 [84]	*N*=202 [55]	*N*=52 [18]
PFS (mo)	4.8 vs 2.5	5.5 vs 2.8	8.3	7.4 vs 4.2	13.7
		HR = 0.44; CI, 0.35–0.55; *P*<0.01			
ORR (CR + PR)	10%^C^	10%^c^	44%^c^	29%^d^	44.2%^c^
					29.9
OS (mo)	NR	17.8 vs 14.3 [83]	NR	NR	
		HR = 0.78; CI, 0.62–0.97; *P*=0.0287			
Study 2			OL	OL	OL vs SOR
			*N*=107 [85]	*N*=31 [81]	*N*=723 [21]
PFS (mo)			8.2	NR	6.7 vs 4.7
					HR = 0.665; CI, 0.522–0.812; *P*=0.0001
ORR (CR + PR)			20%^c^	37%	19.4 vs 9.4%
					*P*=0.0001
OS (mo)			19.8	NR	
TKI failure					
Study 1					OL *N*=62 [19]
PFS (mo)					7.4
ORR (CR + PR)					22.6%^d^
OS (mo)					13.6

^a^Approved in the United States (Note: bevacizumab monotherapy approved only in Europe)

^b^Approved in the United States and Europe

^c^Investigator assessment

^d^Independent assessment

*mRCC* metastatic renal cell cancer, *IFN*-α interferon-α, *PBO* placebo, *PFS* progression-free survival, *HR* hazard ratio, *CI* 95% confidence interval, *ORR* overall response rate, *CR* complete response, *PR* partial response, *OS* overall survival, *NR* not reported, *OL* open-label, *SOR* sorafenib

The newer agent, axitinib, is a potent, selective, second-generation inhibitor of VEGFR-1, 2, and 3 with clinical antitumor activity in a variety of solid tumors [16–20]. In a recent pivotal randomized phase III trial, axitinib demonstrated statistically superior PFS compared with sorafenib, as well as a higher response rate [21]. Although many of the toxicities of axitinib are shared with those of the other TKIs, there are important differences, most notably an apparent higher incidence of hypertension. Moreover, the safety profile for axitinib is distinct from that of sorafenib. Common adverse events (AEs) more frequent with sorafenib versus axitinib were hand-foot syndrome (HFS), rash, alopecia, anemia, hypophosphatemia, hypocalcemia, and elevated lipase whereas the predominant toxicities with axitinib were hypertension, fatigue, nausea, vomiting, and hypothyroidism [21].

Axitinib first demonstrated clinical activity in patients with refractory advanced RCC in a phase II study [18], in which 52 patients with cytokine-refractory mRCC and clear-cell histology received axitinib 5 mg twice daily (BID). An overall response rate of 44% was reported with a median duration of response of 23.0 months (range, 4.2–29.8 months). Median time to progression was 15.7 months (range, 8.4–23.4 months) and median OS was 29.9 months (range, 2.4–35.8 months). In a second phase II trial [19], patients with sorafenib-refractory mRCC received axitinib at a starting dose of 5 mg BID. Axitinib produced a 23% response rate and median duration of response of 17.5 months. Median PFS was 7.4 months (95% CI, 6.7–11.0) and median OS was 13.6 months (95% CI, 8.4–18.8).

In the recent phase III trial in patients with advanced RCC [21], axitinib 5 mg BID demonstrated superior PFS compared with sorafenib 400 mg BID (6.7 versus 4.7 months; *P* = 0.0001) with a significantly higher response rate (19.4 versus 9.4%; *P* = 0.0001) (Table 1). Patient-reported QoL was comparable between the two treatment arms.

## Toxicity profile of new antiangiogenic agents for mRCC

Commonly reported toxicities for antiangiogenic agents in patients with mRCC include class effects of fatigue, asthenia, diarrhea, nausea, anorexia, rash, HFS, and hypertension (Table 2) [21–25]. Other toxicities or combinations of side effects appear to be relatively specific to particular antiangiogenic agents.

**Table 2:** Toxicity profile of targeted therapies as first- and second-line treatment of mRCC

	VEGF inhibitor		TKIs							
	Bevacizumab + IFN-α [23]		Sorafenib [30]		Sunitinib [28]		Pazopanib [55]		Axitinib [21]	
Previous treatment status	Tx-naïve		Cytokine failure		Tx-naïve		Tx-naïve + cytokine failure		TKI + cytokine failure	
Dose modification, % patients										
Dose reduction	40		13		52		36		31	
Dose interruption	–		21		54		42		77	
AE, % patients	AE grade									
	All	3/4	All	3/4	All	3, 4	All	3, 4	All	≥3
Cardiovascular										
Hypertension	26	3	17	4	30	12, 0	40	4, 0	40	16
Constitutional symptoms										
Fatigue	33	12	37	5	54	11, 0	19	2, 0	39	11
Asthenia	32	10	–	–	20	7, <1	14	3, 0	21	5
Hypothyroidism	–	–	–	–	14	2, 0	<10	<1	19	<1
Cutaneous symptoms										
Rash	–	–	40	1	24	1, <1	–	–	13	<1
Hand-foot syndrome	–	–	30	6	29	9, 0	<10	<1	27	5
Mucositis/stomatitis	–	–	–	–	30	1, 0	<10	<1	15	1
Gastrointestinal symptoms										
Diarrhea	20	2	43	2	61	9, 0	52	3, <1	55	11
Nausea	–	–	23	<1	52	5, 0	26	<1, 0	32	3
Vomiting	–	–	16	1	31	4, 0	21	2, <1	24	3
Dyspepsia	–	–	–	–	31	2, 0	–	–	–	–
Anorexia/decreased appetite	36	3	16	<1	34	2, 0	22	2, 0	34	5
Abdominal pain	–	–	11	2	11	2, 0	11	2, 0	–	–
Hemorrhage/bleeding										
All sites	33	3	15	2	–	–	13	–	–	–
Laboratory										
Lymphopenia	–	–	–	13	–	–	31	4, <1	33	3
Neutropenia	7	4	–	–	–	–	34	1, <1	6	1
Thrombocytopenia	6	2	–	–	68	8, 1	32	<1, <1	15	<1
Decreased phosphorus	–	–	–	13	–	–	34	4, 0	13	2
Elevated lipase	–	–	41	12	56	15, 3	–	–	27	5
Anemia/decreased Hb	10	3	8	3	79	6, 2	–	–	35	<1
Proteinuria	18	7	–	–	–	–	<10	<1	–	–

*mRCC* metastatic renal cell cancer, *VEGF* vascular endothelial growth factor, *TKI* tyrosine kinase inhibitor, *IFN*-α interferon-α, *Tx* treatment, *AE* adverse event, *Hb* hemoglobin

### Toxicities across cancer populations

Toxicity profiles of antiangiogenic therapies lack disease specificity and thus can be usefully summarized and compared across disease indications. AEs reported for these agents in patients with mRCC are very similar to toxicities reported for sunitinib in gastrointestinal stromal tumors [22], sorafenib in hepatocellular carcinoma [24], bevacizumab monotherapy in glioblastoma, and bevacizumab plus chemotherapy for metastatic colorectal cancer, non-squamous non–small cell lung cancer, and metastatic breast cancer.

### VEGF inhibitors

Common AEs in patients with RCC receiving bevacizumab/INF-α include pyrexia, anorexia, fatigue, asthenia, bleeding, hypertension, and proteinuria [23]. Bevacizumab is also associated with increased incidence of potentially life-threatening gastrointestinal perforations and thrombovascular events [23, 25]. A meta-analysis of 12,294 patients with a variety of solid tumors treated with bevacizumab in 17 randomized controlled trials reported that the addition of bevacizumab to other cancer therapy increased the risk of gastrointestinal perforation by 1.6- to 5.7-fold, depending on tumor type and dose [26]. In addition, a recent meta-analysis [27] of >10,000 patients with cancer treated with bevacizumab revealed increased incidence of treatment-related mortality, particularly in patients who were also receiving taxanes or platinum agents. In phase III trials of bevacizumab plus INF-α, congestive heart failure (CHF; in <1% patients) and cardiac ischemia/infarction (in 1% of patients) were reported [23, 25].

Specific effects of TKIs commonly include hypertension, HFS (palmoplantar erythrodysesthesia), rash, mucositis, hypothyroidism, and myelosuppression (Table 2).

Across oncology trials with sunitinib, toxicities occurring in ≥20% of patients included anemia, diarrhea, fatigue, nausea, asthenia, mucositis/stomatitis, vomiting, hypertension, HFS, and rash [28]. Sunitinib is also associated with myelosuppression, elevated levels of thyroid-stimulating hormone (TSH), hypothyroidism, and hepatotoxicity including liver failure. In addition, there is increased risk of CHF and decline in left ventricular ejection fraction (LVEF) in 10% of patients [29]. Prolongation of QT interval may also lead to increased risk of ventricular arrhythmias. AEs occurring in ≥20% of sorafenib-treated patients included rash/desquamation, diarrhea, fatigue, HFS, alopecia, and nausea [30]. Sorafenib is also associated with increased risk of life-threatening bleeding. A high frequency of intracerebral hemorrhage has been reported in sorafenib- or sunitinib-treated mRCC patients with brain metastases [31]. Pazopanib is associated with hypothyroidism and proteinuria, as well as having variable effects on glucose levels [32]. Pazopanib can also cause hepatotoxicity; monitoring of liver function is required and dose reduction may be necessary in patients with baseline elevation in total bilirubin and other hepatic function tests [32]. Similar associations have been observed with sorafenib, with dose reductions suggested for patients with hepatic dysfunction [33]. Hyperglycemia has been reported in 41% of pazopanib-treated versus 33% of placebo-treated patients, whereas hypoglycemia was reported in 17% of pazopanib- versus 3% of placebo-treated patients.

Toxicities of concern reported for some of the investigational TKIs include cholecystitis and gall bladder enlargement with motesanib, proteinuria with axitinib, and mucositis with XL184. There appear to be some relative safety differences across the various VEFG-inhibitor therapies, although the data must still be considered incomplete at this time. In particular, bevacizumab is associated with a low incidence of hypothyroidism, sorafenib has low cardiac toxicity compared to sunitinib, and recipients of pazopanib report less fatigue.

### Proposed mechanisms of common toxicities

#### Hypertension

Hypertension occurs in 17% to 45% of TKI-treated patients with RCC, with grade 3 or 4 hypertension reported in 3% to 16% of patients. Elevated blood pressure (BP) typically presents early, within 3 to 4 weeks of treatment initiation [34, 35]. Some studies of TKI-mediated BP effects reported elevations as early as the first day [36] to first week [37] of treatment.

The exact mechanisms underlying VEGF/VEGFR inhibitor–associated hypertension remain unknown but increased BP, a dose-dependent effect of these inhibitors, is believed to be caused by increases in vascular tone and peripheral resistance. Interestingly, emergence of hypertension with these agents, including axitinib, may serve as a biomarker for antitumor efficacy [38–40].

In the sorafenib-refractory study of axitinib [19], peripheral edema and hypertension were reported by 19.4% and 45.2% of patients, respectively. Hypertension remains the major cardiovascular-related toxicity of axitinib, reported in 51% of patients [18, 19]. A pooled analysis of phase II studies of axitinib in mRCC [40] reported that patients with at least one diastolic BP (dBP) measurement ≥90 mmHg during treatment had a significantly longer median OS compared with patients with dBP <90 mmHg (30.1 versus 10.2 months, respectively; *P* < 0.001). Likewise, an analysis of sunitinib clinical trials in patients with mRCC [39], showed that treatment-emergent hypertension was an independent predictor of PFS and OS (*P* < 0.001). PFS was 12.5 versus 2.5 months in patients with maximal systolic BP (sBP) ≥140 mmHg versus <140 mmHg, respectively (*P* < 0.001). Similarly, significant clinical benefit was reported for dBP ≥90 mmHg compared with <90 mmHg. Effective control of BP with antihypertensive treatment did not affect the improved clinical outcome. Currently, a randomized prospective phase II axitinib trial in patients with mRCC is evaluating axitinib-related dBP changes as a possible predictive biomarker for response (ClinicalTrials.gov identifier: NCT00835978).

Before starting TKI therapy, BP should be controlled for approximately 1 week. Hypertension should be monitored and controlled with appropriate antihypertensive agents, with weekly monitoring of BP during the first cycle and 2 to 3 weeks thereafter until a stable BP has been reached, and then monitored per standard medical practice [41]. Likewise, BP should be monitored following discontinuation of TKI therapy since BP can drop rapidly.

Patients who develop stage I hypertension (≥140/90 mmHg) or have increases in dBP ≥20 mmHg from baseline should initiate antihypertensive therapy, modify the dose of the current agent for better control, or add a second antihypertensive agent [41]. In some instances, dose reduction of the TKI inhibitor can be implemented to manage TKI-induced hypertension. The major classes of antihypertensive agents, including angiotensin-converting enzyme (ACE) inhibitors, beta blockers, and calcium channel blockers, have been used to treat TKI-induced hypertension. There are no consensus recommendations, however, for the use of specific antihypertensive agents in this setting [42]. Antihypertensive agents should be individualized to suit the patient’s clinical status. ACE inhibitors, for example, are preferred for patients with proteinuria, chronic kidney disease risks, or metabolic syndrome [42].

#### Cutaneous reactions

Rash, HFS, and mucositis/stomatitis are common effects of antiangiogenic agents. HFS is characterized by palmoplantar lesions in areas of friction or trauma, commonly in the hands and feet. HFS may significantly affect a patient’s QoL and physical functioning and often leads to treatment modification or discontinuation [30, 43]. The precise mechanisms causing these events are largely unknown. In a sunitinib study, skin toxicity appeared after 3 to 4 weeks of treatment and was characterized by dermal vascular modifications, scattered keratinocyte necrosis, and intra-epidermal cleavage, which may be mediated via direct anti-VEGFR and/or PDGF receptor effects on dermal endothelial cells [44].

#### Hypothyroidism

Antiangiogenic agents are known to affect thyroid homeostasis but the precise mechanisms are not well understood. Biochemical and clinical hypothyroidism is commonly reported in patients with RCC receiving sunitinib and sorafenib [45–47]. An increase in TSH and decreases in thyroid hormone, indicative of hypothyroidism, has been reported in sunitinib-treated patients with gastrointestinal tumors [48]. VEGFR inhibitors such as sunitinib may induce thyroiditis and hypothyroidism via a direct effect on the thyroid through inhibition of VEGFR [49]. Thyroid dysfunction may also result from regression of capillaries around thyroid follicles due to VEGFR inhibition [50]. Changes in TSH appeared to correlate with fatigue in patients receiving axitinib [51]. Therefore, thyroid-function monitoring is recommended with management of hypothyroidism following standard guidelines for levothyroxine replacement therapy [52].

#### Fatigue

Fatigue is experienced by 19% to 77% of patients receiving antiangiogenic agents. The most common factors contributing to fatigue in patients with cancer independent of treatment with angiogenesis inhibitors are hypothyroidism, anemia, and dehydration. Hypogonadism may also contribute to the fatigue associated with sunitinib and sorafenib [53]. Fatigue has a high impact on patient QoL and should be monitored closely, following appropriate treatment guidelines to alleviate symptoms [54].

#### Gastrointestinal disturbance

Gastrointestinal AEs in patients with RCC treated with antiangiogenic agents include diarrhea, nausea, and vomiting (Table 3). These AEs are usually not associated with treatment discontinuation because of successful management by standard medical interventions such as antidiarrheal medications and dietary modification.

**Table 3:** Toxicity profile of axitinib in phase II studies

	mRCC [19] *N* = 62		mRCC [18] *N* = 52		TC [16] *N* = 60		NSCLC [20] *N* = 32		Melanoma [63] *N* = 32	
Previous treatment status	Sorafenib		Cytokine		131 Iodine					
AE, % patients	AE grade									
	All	3/4	All	3/4	All	≥3	All	3	All	>3
Fatigue	77	16	52	8	50	5	50	22	63	22
Diarrhea	61	15	60	10	48	3	41	3	31	0
Anorexia	48	0	35	2	30	0	50	0	–	–
Hypertension	45	16	58	14	28	12	22	9	44	6
Nausea	44	7	44	0	33	0	34	0	22	0
Dyspnea	39	15	–	–	–	–	–	–	–	–
Dysphonia	37	0	19	0	–	–	28	0	34	0
Hand-foot syndrome	36	16	8	–	15	0	–	–	–	3
Mucosal inflammation	34	2	–	–	–	–	16	0	16	0
Vomiting	32	5	21	0	13	0	19	3	–	–
Weight decrease	31	5	27	0	25	3	16	0	16	0
Cough	29	0	–	–	–	–	–	–	–	–
Headache	29	2	15	0	22	3	–	–	–	–
Arthralgia	27	3	14	2	–	–	22	0	19	6
Constipation	26	0	14	0	–	–	–	–	–	–
Dysgeusia	23	0	12	0	–	–	–	–	–	–
Abdominal pain	21	11	12	0	–	–	–	–	–	–
Pain in extremity	21	3	19	4	–	–	–	–	16	0
Stomatitis	–	–	17	2	25	0	–	–	16	3
Proteinuria	–	–	8	0	18	5	–	–	38	3
Rash	–	–	–	–	15	0	16	0	–	–

*mRCC* metastatic renal cell cancer, *TC* thyroid cancer, *NSCLC* non–small cell lung cancer, *AE* adverse event

#### Cardiovascular toxicities

Cardiovascular toxicities of TKIs include hypertension, peripheral edema, and cardiac dysfunction [28, 30, 55]. The rate of TKI-associated cardiovascular toxicities is not well established. Cardiac damage is manageable, provided the patients receive appropriate cardiac monitoring and treatment at the first indication of myocardial damage [56]. Monitoring for drug-related toxicities can be challenging, as symptoms such as dyspnea, chest, pain, and dizziness can be ambiguous disease indicators in patients with advanced cancer. The use of beta blockers such as carvedilol and drugs such as simvastatin has been suggested as a means to protect against TKI-induced cardiac toxicities [56]. Importantly, decline in LVEF has preceded CHF in sorafenib- and sunitinib-treated patients, mainly in those with a history of coronary artery disease. LVEF declines have been observed in patients with mRCC treated with sunitinib, but it is not known if patients with cardiac conditions have a greater chance of developing sunitinib-related LVEF [57]. Baseline and periodic assessment of LVEF are strongly recommended for patients receiving TKI therapy. Special emphasis must be placed on monitoring for the clinical signs and symptoms of CHF. Patients with signs and symptoms of CHF should be thoroughly evaluated (including LVEF assessment) and discontinue therapy. Physicians are advised to consider carefully the cardiac risk: benefit ratio for any patient before initiating therapy with VEGF inhibitors.

#### Proteinuria

Proteinuria is mostly observed in patients receiving bevacizumab (Table 2). The mechanism underlying proteinuria is unclear but it may reflect a role for VEGF in normal glomerular endothelial repair [58]. Patients should be monitored for proteinuria before and after treatment. Therapy should be discontinued in patients with grade 4 proteinuria.

#### Bleeding and wound healing

Bleeding, including epistaxis, hematemesis, gastric bleeding, and brain hemorrhage, is associated with VEGF inhibitors and is more common with bevacizumab [59]. While bleeding is generally manageable, it can be serious and sometimes fatal. Patients with serious bleeding should not receive bevacizumab. Angiogenesis is required for wound healing and, thus, anti-VEGF agents may directly affect the healing process. Wound-healing complications, such as slow or incomplete healing following surgery, have been reported for bevacizumab and pazopanib. These events were fatal in some cases.

#### Thromboembolic events

Angiogenesis inhibition, as well as cytotoxic chemotherapy, is associated with increased risk of both arterial thromboembolic events (ATE) and venous thromboembolic events (VTE) [60]. Several factors related to VEGF inhibition are believed to contribute to the increased risk of ATE and VTE, including the role of VEGF in the regeneration of endothelial cells. A pooled analysis of clinical trials, including trials in mRCC, reported that bevacizumab was significantly associated with an increased risk of developing VTE in patients with cancer [61]. In this analysis, the incidence of all-grade and high-grade VTE was 11.9% and 6.3%, respectively. A recent meta-analysis to assess the risk of ATE reported that treatment with sunitinib and sorafenib is associated with a three-fold increase in the risk of ATE, with an overall incidence of 1.3% in patients with RCC [62]. Myocardial infarction and cardiac ischemia have also been reported for sunitinib and sorafenib.

### Follow-up

Careful evaluation and follow-up of reported toxicities and their response to management often allow patients to continue treatment safely on the prescribed effective doses of antiangiogenic agents. AEs leading to dose interruption or reduction should be closely monitored so therapy can be reinstituted once side effects improve or resolve.

## Axitinib

### Axitinib-related toxicities in advanced RCC

#### Common toxicities

AEs associated with axitinib including a higher incidence of hypertension compared with some of the other TKIs, generally respond to supportive measures and dose modifications. The most common axitinib-related AEs reported across phase II trials [16, 18–20, 63] were fatigue, diarrhea, hypertension, and anorexia (Table 3). The most common grade 3/4 AEs were hypertension, fatigue, and diarrhea. The most commonly reported hematologic AE was grade 1/2 anemia, which did not require dose reduction or interruption [18, 20].

In the phase III study of axitinib versus sorafenib [21], common AEs more frequently reported with sorafenib versus axitinib, respectively, included anemia (52% versus 35%), HFS (51% versus 27%), rash (32% versus 13%), and alopecia (32% versus 4%) and AEs more frequently occurring with axitinib versus sorafenib, respectively, included hypertension (40% versus 29%), fatigue (39% versus 32%), nausea (32% versus 22%), vomiting (24% versus 17%), and hypothyroidism (19% versus 8%). The incidence of diarrhea was similar for axitinib and sorafenib (55% and 53%, respectively). Axitinib does not appear to cause neutropenia and thrombocytopenia, which have been reported with sunitinib.

Axitinib toxicities are very similar and manageable in patients with cancers other than RCC. For example, in the phase II study of axitinib in 60 patients with advanced thyroid cancer refractory to conventional therapy, grade ≥3 treatment-related AEs were hypertension (12%), fatigue (5%), proteinuria (5%), and diarrhea, headache, and weight decrease (3% each) [16].

#### Dose-limiting toxicities

Dose-limiting AEs leading to axitinib dose reduction or interruption include hypertension, fatigue, and diarrhea. In a phase I study of patients receiving 5 to 30 mg axitinib BID [64], hypertension was the primary dose-limiting toxicity. One patient receiving axitinib (20 mg BID reduced to 10 mg BID) died acutely with grade 4 hemoptysis. In patients receiving the recommended phase II 5-mg BID dose, dose-limiting toxicities were grade 2 stomatitis and grade 3 diarrhea (*n* = 1 each). In phase II studies [18, 19], common AEs leading to axitinib dose interruption were dyspnea, nausea, fatigue, hypertension, and vomiting. In the sorafenib-refractory mRCC study with axitinib [19], AEs led to study discontinuation in 19% of patients and to temporary dose interruptions or reductions in 73% and 45% of patients, respectively. In the cytokine-refractory mRCC study of axitinib [18], 15 patients (28.8%) had a dose reduction due to AEs. Dose reduction was required for grade 3 diarrhea and fatigue (*n* = 2 each); gastrointestinal upset, dehydration, myalgia, and gout (*n* = 1 each); and grade 2 hypertension (*n* = 7). In this latter study, some patients took axitinib continuously for up to 5 years without evidence for cumulative toxicities.

### Management of axitinib-related toxicities in advanced RCC

#### Assessments and monitoring of toxicities

Pretreatment assessment should be performed with particular attention to the presence of comorbidities (e.g., preexisting hypertension) that may indicate more frequent monitoring and anticipation of possible dose reductions. Patients with preexisting cardiovascular dysfunction and cardiac risk factors should be monitored regularly with BP assessment at baseline and during treatment. Thyroid profiles should be assessed at baseline and every 2 to 3 months after initiation of therapy [65]. Very rarely, high hematocrits have been seen with axitinib [18, 66] and should be treated appropriately.

### Management of common axitinib toxicities

Prevention and management strategies for axitinib-related AEs are presented in Table 4 and discussed below.

**Table 4:** Prevention and management axitinib-related adverse events

	Prevention	Grade ≥1	Grade 3 or 4
Skin			
Hand-foot syndrome [65, 73]	Routine manicure/pedicure		Delay or adjust dose
	Remove calluses with proper tools		Implement topical treatment
	Cushion pressure points and protect areas		
	Avoid constriction/friction in concerned areas		
	Use moisturizer (alcohol-free) after bathing		
Oral mucositis/stomatitis [65]	Maintain oral hygiene	Use mucosal-covering agents	Dose reduction or interruption
	Use salt and baking soda mouthwash	Use topical lidocaine solutions	
	Consume a soft diet	If oral candidiasis present:	
		use oral fluconazole or local clotrimazole troche	
Rash [73]	Use moisturizer twice daily (alcohol-free)	Topical hydrocortisone cream 1%	Oral prednisone
	Avoid abrasive clothing/detergents/shampoo		For grade 4, provide referral to specialist
	Avoid direct sunlight		
	Use SPF 30 lotion/clothing		
Gastrointestinal			
Diarrhea [69]	Avoid diarrhea-enhancing foods/drinks/supplements (e.g., lactose, alcohol, caffeine, fiber)	Loperamide or diphenoxylate	Admit patient to hospital
	Drink 8–10 glasses of clear liquids daily		
Nausea/Vomiting [70–72]	Eat small meals frequently	Metoclopramide, prochlorperazine, or haloperidol; add ondansetron or granisetron	
	Sip fluids steadily		
Anorexia [86]		Consider megestrol acetate	
Constitutional			
Fatigue [65, 86]	Monitor fatigue levels	Exclude/treat contributing conditions (e.g., depression, hypothyroidism, pain, anemia)	Dose reduction or interruption
	Use energy-conserving strategies	Provide supportive care	
	Use distraction strategies	Consider psychostimulant (e.g., methylphenidate, modafanil)	

*SPF* sun protective factor

#### Fatigue

Fatigue (all grades) with axitinib treatment occurs in 39% of patients [21]. Treatment of fatigue is supportive in nature, requiring a thorough assessment of other possible exacerbating factors (e.g., sleep disturbance, comorbid conditions, concomitant medications, hypothyroidism, or anemia). Supportive strategies include a range of approaches, from decreasing energy expenditure to psychosocial interventions. Pharmacologic strategies include treating contributing factors (e.g., anemia, hypothyroidism) and the judicious use of psychostimulants, including methylphenidate and modafanil [67]. Axitinib-induced thyroid dysfunction and hypothyroidism are easily controlled by thyroid hormone replacement therapy [68].

#### Gastrointestinal symptoms

Over half of patients will experience axitinib-related gastrointestinal disturbances. Patients should be advised to consume frequent small meals, drink clear fluids in regular small amounts, and avoid foods or drinks that may exacerbate diarrhea (such as dairy products) [69–72]. Diarrhea can be controlled with the use of standard antidiarrheal medication and proper hydration.

#### Hand-foot syndrome

HFS is experienced by up to 36% of axitinib-treated patients and can be minimized by various skincare measures prior to initiating antiangiogenic therapy [65, 73]. Although severe episodes of HFS may necessitate dose alterations, topical treatments and avoidance of friction, especially in the feet, may provide some relief. In one study, patients treated with topical urea or tazarotene or a two-agent combination (e.g., urea, fluorouracil, and/or tazarotene) reported improvement in HFS symptoms resulting from TKI therapy [74].

#### Hypertension

Hypertension should be controlled before starting antiangiogenic therapy. BP ≥140/90 mmHg should be actively treated using standard antihypertensive medications. Reaching a target <140/90 mmHg may require different combinations of standard agents and a different BP goal may be appropriate for some patients.

For hypertensive patients with uncontrolled BP, the dose of antihypertensive medication should be increased or, if on a maximal dose, a second agent should be added and its dose increased as appropriate. At pretreatment assessment, hypertensive patients can initiate therapy with short-acting agents to quickly achieve BP control before exchanging medications for longer-acting agents. This approach allows cancer therapy to begin expeditiously. BP assessment prior to initiating therapy, every 2 weeks during the first 3 months of therapy, and monthly thereafter is recommended. Patients should also monitor their BP daily at home (prior to taking axitinib). Axitinib-treated patients with sBP >150 mmHg or dBP >100 mmHg or experiencing symptoms such as headache or visual disturbance indicating hypertension should promptly contact their physician for axitinib-dose modification.

#### Drug-drug interactions

Axitinib is primarily metabolized in the liver by the cytochrome P450 (CYP) 3A4 isozyme and to a lesser extent by CYP2C19 and CYP1A2. Less than 1% of the administered dose is excreted in the urine unchanged [64]. Both inducers and inhibitors of CYP metabolism may affect axitinib plasma exposures. Therefore, concomitant use of known potent CYP3A4 inhibitors (i.e., grapefruit juice, verapamil, ketoconazole, miconazole, erythromycin, telithromycin, clarithromycin), as well as CYP3A4 or CYP1A2 inducers (e.g., carbamazepine, dexamethasone, phenobarbital, phenytoin), should be avoided in patients receiving axitinib. Combination therapies with agents such as 5-fluorouracil, cisplatin, carboplatin, docetaxel, and paclitaxel did not affect the pharmacokinetic profile of axitinib.

### Axitinib dose modification

Dose modification or treatment interruption may be required to alleviate axitinib-related toxicities. Stepwise increases from the starting dose (5 mg BID) to 7 mg BID and then 10 mg BID may be instituted at 2-week intervals in the absence of grade ≥3 AEs or the development of hypertension. The benefit of titrating to higher doses is supported by preliminary data in RCC in which higher plasma axitinib exposure was associated with improved outcomes [75, 76]. Dose reductions are also implemented in a stepwise fashion. Thus, 5 mg BID is reduced to 3 mg BID, and then to 2 mg BID, if needed. Similarly, for patients receiving 7 or 10 mg BID, stepwise reduction should be to the next lowest dose. Recommendations for dose modifications in patients who develop hypertension or proteinuria are presented in Table 5. Dose modifications for other nonhematologic and hematologic events are presented in Table 6.

**Table 5:** Axitinib modification for hypertension and proteinuria [19]

Adverse event	Action
Hypertension	
sBP >150 mmHg or dBP >100 mmHg (Two readings at least 1 h apart)	*Previously normotensive:* Initiate anti-HTN therapy; maintain axitinib dose
	*Previously HTN:* Increase dose of anti-HTN therapy; if already on maximal dose, reduce axitinib dose by one level
sBP >160 mmHg or dBP >105 mmHg (Two readings at least 1 h apart)	Interrupt axitinib treatment^a^; adjust dose of anti-HTN agents until BP <150/100 mmHg; immediately restart axitinib treatment at one lower-dose level
Recurrent following dose reduction (Two readings at least 1 h apart)	Reduce axitinib dose further by one level
Proteinuria	
Proteinuria >1 g/24 h	Perform 24-h urine collection; continue axitinib dose while awaiting test results
Proteinuria ≥2 g/24 h	Interrupt axitinib treatment; wait until daily protein excretion is <2 g; restart axitinib treatment at a same dose or reduce by one dose level

^a^Patients should be closely monitored for the development of hypotension

*sBP* systolic blood pressure, *dBP* diastolic blood pressure, *HTN* hypertensive, *BP* blood pressure

**Table 6:** Axitinib dose modification by grade of adverse event^a^

AE grade	Type	Modification
1	Non-hematologic or Hematologic	Continue same dose
2	Non-hematologic or Hematologic	Continue same dose
3	Non-hematologic	Decrease dose to one lower-dose level
	Hematologic	Continue same dose
4	Non-hematologic or hematologic^b^	Interrupt dosing; restart at one lower-dose level when AE improves to CTCAE grade 2 or better

^a^Not including hypertension or proteinuria (see Table 3)

^b^Grade 4 lymphopenia or asymptomatic biochemistry abnormality may continue without interruption

*AE* adverse event, *CTCAE* Common Terminology Criteria for Adverse Events

## Conclusions

The new generation of targeted therapies for advanced RCC offers significant benefit compared with prior approaches such as cytokines and chemotherapy. However, significant potential for a different spectrum of toxicities clearly exists with these newer agents, including those targeting angiogenesis. Class-effects such as hypertension, fatigue, and gastrointestinal disturbances are common with all the antiangiogenic agents and should be anticipated and proactively managed. Other unique but important toxicities, including hypothyroidism, proteinuria, cutaneous reactions, and hemorrhage, occur less often. The mechanisms underlying the toxicities are beginning to be revealed, but considerable research in this area is needed. This understanding could lead to new therapies with improved toxicity profiles and/or greater specificity for selected subtypes of RCC.

Emerging evidence suggests that certain adverse effects may be biomarkers for efficacy in RCC. Despite a lack of complete understanding of the underlying biological mechanisms, selected toxicities such as hypertension may prove to be clinically useful surrogates of response if they are reproducible and correlate well with outcomes. Ongoing pharmacogenomic research is focused on identifying specific gene polymorphisms that may be associated with increased toxicity or improved outcomes with RCC therapies.

Proper management of these AEs will ensure that patients receive optimal benefit from these newer therapies. In addition, grade 1 and 2 toxicities should not be overlooked when treating patients since these can be challenging for patients who must take drugs on a daily basis, can have substantial effects on QoL and overall healthcare costs, and may lead to treatment discontinuations. The potentially significant impact of these cumulative low-grade AEs on patients must also be weighed against the marginal clinical benefit observed with certain targeted agents in unselected patient populations. Fojo and Parkinson [77] have suggested that identification of patient subsets by use of clinically validated biomarkers, developed in parallel with new targeted therapy, may inform more biologically based patient selection. This approach offers the potential in the future of maximizing efficacy, minimizing toxicity and effects on QoL, and reducing cost.

Proactive management of these toxicities involves routine monitoring of clinical symptoms, BP, and laboratory parameters, coupled with early intervention. Active and early treatment of adverse effects is vital to maintain treatment and limit the need for dose reductions, interruptions, or discontinuations. Successful planning to anticipate the occurrence of toxicities and effective management will help ensure that patients with RCC receiving targeted therapies such as axitinib have optimal outcomes with AEs that are infrequent, low-grade, and manageable.
